# Neurological manifestations of COVID-19: a systematic review

**DOI:** 10.1186/s13054-020-03121-z

**Published:** 2020-07-13

**Authors:** Gaurav Nepal, Jessica Holly Rehrig, Gentle Sunder Shrestha, Yow Ka Shing, Jayant Kumar Yadav, Rajeev Ojha, Gaurab Pokhrel, Zhi Lan Tu, Dong Ya Huang

**Affiliations:** 1 Maharajgunj Medical Campus, Tribhuvan University Institute of Medicine, Kathmandu, Nepal; 2 University of New England College of Osteopathic Medicine, Biddeford, ME USA; 3 Department of Anesthesiology, Tribhuvan University Teaching Hospital, Kathmandu, Nepal; 4 National University Hospital, Singapore, Singapore; 5 Department of Neurology, Tribhuvan University Teaching Hospital, Kathmandu, Nepal; 6 Department of Urology, Tongji Hospital of Tongji Medical College, Huazhong University of Science and Technology, Wuhan, Hubei China; 7 Department of Neurology, Shanghai Pudong Hospital, Fudan University Pudong Medical Center, Shanghai, China; 8 Department of Neurology, Shanghai East Hospital of Tongji University School of Medicine, Shanghai, China

**Keywords:** COVID-19, SARS-CoV-2, Neurological manifestation, Brain

## Abstract

**Introduction:**

Severe acute respiratory syndrome coronavirus 2 (SARS-CoV-2) is responsible for the global spread of coronavirus disease (COVID-19). Our understanding of the impact this virus has on the nervous system is limited. Our review aims to inform and improve decision-making among the physicians treating COVID-19 by presenting a systematic analysis of the neurological manifestations experienced within these patients.

**Methods:**

Any study, released prior to May 20, 2020, that reported neurological manifestations in patients infected by SARS-CoV-2 was systematically reviewed using the PRISMA (Preferred Reporting Items for Systemic review and Meta-Analysis) statement.

**Results:**

Our systematic review included data from 37 articles: twelve retrospective studies, two prospective studies, and the rest case reports/series. The most commonly reported neurological manifestations of COVID-19 were myalgia, headache, altered sensorium, hyposmia, and hypogeusia. Uncommonly, COVID-19 can also present with central nervous system manifestations such as ischemic stroke, intracerebral hemorrhage, encephalo-myelitis, and acute myelitis, peripheral nervous manifestations such as Guillain-Barré syndrome and Bell’s palsy, and skeletal muscle manifestations such as rhabdomyolysis.

**Conclusion:**

While COVID-19 typically presents as a self-limiting respiratory disease, it has been reported in up to 20% of patients to progress to severe illness with multi-organ involvement. The neurological manifestations of COVID-19 are not uncommon, but our study found most resolve with treatment of the underlying infection. Although the timeliness of this review engages current challenges posed by the COVID-19 pandemic, readers must not ignore the limitations and biases intrinsic to an early investigation.

## Introduction

Coronavirus disease (COVID-19) is caused by the novel virus, severe acute respiratory syndrome coronavirus 2 (SARS-CoV-2) [1]. Since its recent discovery in Wuhan, China, coronavirus disease has spread across the world, leaving physicians challenged by its variable clinical manifestations.

Most patients infected by SARS-CoV-2 have presented with a mild clinical course: beginning with fever and dry cough, progressing to a form of mild or moderate respiratory disease, and resolving without specific treatment [2]. Serious complications of the infection, however, remain a central concern. Acute respiratory distress syndrome, acute heart injury or failure, acute kidney injury, sepsis, disseminated intravascular coagulation, and life-threatening metabolic derangements have all been reported in COVID-19 patients, particularly among those with underlying comorbidities or advanced age [1, 3].

As knowledge of SARS-CoV-2 and its clinical appearance continue to grow, the literature has shown a significant number of infected patients exhibit neurological symptoms [4, 5]. In this systematic review, we evaluate various neurological manifestations reported in COVID-19 patients and hypothesize their underlying pathophysiology. We deem the timeliness of this systematic review relevant, given the state of the COVID-19 pandemic, but encourage readers to consider the implications of early review and analysis in the clinical setting.

## Methods

Our systematic review utilized the PRISMA (Preferred Reporting Items for Systemic review and Meta-Analysis) statement in conjunction with the PRISMA checklist and flow diagram for manuscript format development [6].

### Literature search

The following databases were reviewed for published studies prior to May 20, 2020: PubMed, Google Scholar, and China National Knowledge Infrastructure (CKNI). We also searched pre-print servers including Research square, medRxiv, SSRN, and ChinaXiv. Boolean logic was used for conducting database search and Boolean search operators “AND” and “OR” were used to link search terms. The following search strategy was adopted: COVID-19 OR SARS-CoV-2 OR 2019-nCoV OR nCoV OR novel corona AND neurological OR neurologic OR brain OR CNS OR nervous AND manifestation OR symptoms OR presentation. Titles, abstracts, and full text were screened to ensure they met eligibility criteria. Two authors (GN and JHR) screened, retrieved, and excluded reports. Additional investigators were consulted if uncertainty arose during the review process.

### Eligibility criteria

We included any study, published in any language, which reported neurological manifestations in patients infected by SARS-CoV-2. This included case reports and pre-print publications. We excluded all review articles, hypotheses papers, and papers reporting neurological symptoms in MERS-CoV and SARS-CoV patients.

### Data extraction

Data was manually extracted from eligible studies by the research investigators. The following variables were included: first author, type of design, site of study, year of publication, published journal or pre-print server, sample size, and reported neurological manifestations.

### Outcome measures

Our outcome was to elucidate the neurological manifestations of COVID-19 reported in the medical literature. The results were divided into three categories: central nervous system manifestations (e.g., headache, encephalopathy, and stroke), peripheral nervous system impairment (e.g., dysfunction of taste, dysfunction of smell, neuropathy), and skeletal muscle manifestations (e.g., myalgia).

## Results

### Study characteristics

**Fig. 1:**
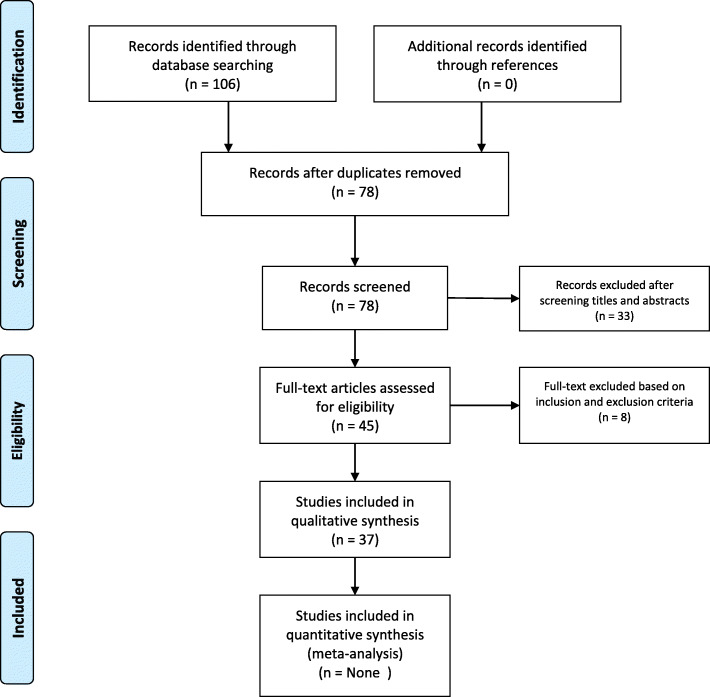
PRISMA flow diagram depicting the flow of information through the different phases of a systematic review

In total, our literature search yielded 106 articles. After excluding duplicates and those not meeting inclusion criteria, 37 papers were included in our systematic review. Figure 1 displays the results of our literature search and selection. The characteristics of each study are summarized in Table 1. There were twelve retrospective studies [1, 2, 5, 7, 18–23, 35, 40], two prospective studies [36, 37], and the rest were case reports/series. One article was a multicenter study [36], 18 were from mainland China, six from the USA [11, 15, 30, 34, 35, 39], five from Iran [14, 17, 33, 37, 38], four from Italy [8, 23, 29, 32], and one each from Japan [24], Switzerland [31], and Spain [10]. Out of all included studies, one was published in a premier news agency of China [12], eight were unpublished scientific articles deposited in pre-print servers [7, 13, 16, 26, 28–31], and the remaining 28 were journal publications.

**Table 1:** Characteristics of studies included in our systematic review

**SN**	**Study**	**Type of publication**	**Country**	**Source**	**Neurological manifestation**
1	Mao 2020 [5]	Retrospective study	China	JAMA Neurology	Total patients: 214 Any: 78 (36.44%) CNS: 53 (24.76%) • Dizziness: 36 (16.82%) • Headache: 28 (13.08%) • Impaired consciousness: 16 (7.4%) • Acute cerebrovascular disease: 6 (2.8%) • Ataxia: 1 (0.5%) • Epilepsy: 1 (0.5%) PNS: 19 • Hypoguesia: 12 (5.6%) • Hyposmia: 11 (5.14%) • Neuralgia: 5 (2.33%) Muscle injury: 23 (10.74%)
2	Li 2020 [7]	Retrospective study	China	SSRN (preprint)	Total patients: 224 • Acute ischemic stroke: 11 (5%) • Cerebral venous sinus thrombosis: 1 (0.5%) • Intracerebral hemorrhage: 1 (0.5%)
3	Toscano 2020 [8]	Case-report/series	Italy	The New England Journal of Medicine	Guillain-Barré syndrome
4	Zhao Hua 2020 [9]	Case-report/series	China	The Lancet Neurology	Guillain-Barré syndrome
5	Gutiérrez-ortiz 2020 [10]	Case-report/series	Spain	Neurology	Miller Fisher syndrome and polyneuritis cranialis
6	Poyiadji 2020 [11]	Case-report/series	USA	Radiology	Encephalitis
7	Huaxia 2020 [12]	Newspaper article	China	Xinhua News	Encephalitis
8	Zhai 2020 [13]	Case-report/series	China	Research square (preprint)	Ischemic stroke
9	Sharifi-Razavi 2020 [14]	Case-report/series	Iran	New Microbes and New Infections	Intra-cerebral hemorrhage
10	Filatov 2020 [15]	Case-report/series	USA	Cureus	Encephalopathy
11	Zhao Kang 2020 [16]	Case-report/series	China	medRxiv (preprint)	Acute myelitis
12	Karimi 2020 [17]	Case-report/series	Iran	Iran Red Crescent Medical Journal	Encephalopathy
13	Chen Tao 2020 [18]	Retrospective study	China	BMJ	Total: 274 patients • Myalgia: 60 • Headache: 31 • Hypoxic encephalopathy: 24
14	Qiu 2020 [19]	Retrospective study	China	The Lancet Infectious disease	Total: 36 patients • Headache: 3 patients
15	Zhou 2020 [20]	Retrospective study	China	The Lancet	Total: 191 patients • Myalgia: 29 patients
16	Guan 2020 [1]	Retrospective study	China	The New England Journal of Medicine	Total: 1099 patients • Headache: 150 patients • Myalgia: 164
17	Chen Nanshan 2020 [21]	Retrospective study	China	The Lancet	Total: 99 patients • Headache: 8 • Myalgia: 11 • Confusion: 9
18	Huang 2020 [2]	Retrospective study	China	The Lancet	Total: 41 • Myalgia: 18 • Headache: 3
19	Wang 2020 [22]	Retrospective study	China	JAMA	Total: 138 • Myalgia: 48 • Headache: 9
20	Giacomelli 2020 [23]	Retrospective study	Italy	Clinical Infectious Diseases	Smell/taste disorder
21	Moriguchi 2020 [24]	Case-report/series	Japan	International Journal of Infectious Diseases	Meningitis/encephalitis
22	Jin 2020 [25]	Case-report/series	China	Emerging Infectious Diseases	Rhabdomyolysis
23	Fu 2020 [26]	Case-report/series	China	Research square (preprint)	Two cases of ischemic stroke
24	Ye 2020 [27]	Case-report/series	China	Brain, Behavior, and Immunity	Encephalitis
25	Wan 2020 [28]	Case-report/series	China	Research Square (preprint)	Bell’s palsy
26	Pilotto 2020 [29]	Case-report/series	Italy	medRxiv (preprint)	Encephalopathy
27.	Zhang 2020 [30]	Case-report/series	USA	medRxiv (preprint)	Acute disseminated encephalomyelitis
28.	Bernard-valnet 2020 [31]	Case-report/series	Switzerland	medRxiv (preprint)	Encephalitits
29.	Padroni 2020 [32]	Case-report/series	Italy	Journal of Neurology	Guillain-Barré syndrome
30.	Sedaghat 2020 [33]	Case-report/series	Iran	Journal of Clinical Neuroscience	Guillain-Barré syndrome
31.	Virani 2020 [34]	Case-report/series	USA	ID Cases	Guillain-Barré syndrome
32.	Yan 2020 [35]	Retrospective study	USA	International Forum of Allergy & Rhinology	Smell/taste disorder
33.	Lechien 2020 [36]	Prospective study	Multicentre study in Europe	European archives of oto-rhino-laryngology	Smell/taste disorder
34.	Moein 2020 [37]	Prospective study	Iran	International Forum of Allergy & Rhinology	Smell/taste disorder
35.	Haddadi 2020 [38]	Case-report/series	Iran	Cureus	Intracerebral hemorrhage
36.	Oxley 2020 [39]	Case-report/series	USA	The New England Journal of Medicine	Ischemic stroke
37.	Lu 2020 [40]	Retrospective study	China	Epilepsia	Total patients: 304 • Encephalopathy: 8

### Central nervous system manifestations

#### Headache

**Fig. 2:**
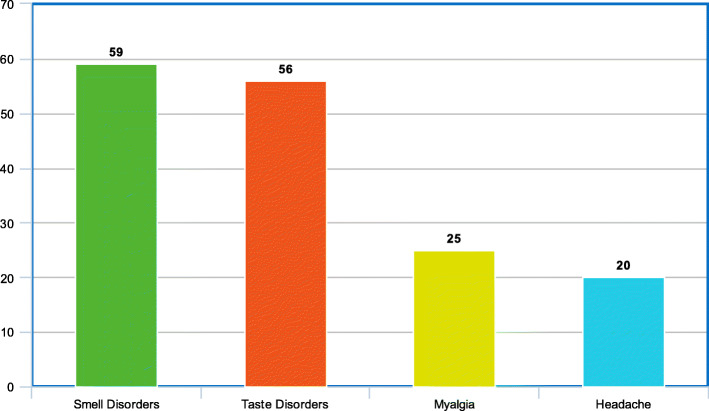
Common neurological manifestations of COVID-19 expressed as percentage

Eight retrospective studies reported COVID-19 patients presenting with headache. We found that an overall average of 19.88% of patients experienced headache (Fig. 2). Mao et al. reported that some of COVID-19 patients with neurologic manifestation initially presented with fever and headache only. However, several days later, they developed cough, throat pain, lymphopenia, and a ground-glass appearance on their respective chest computed tomography (CT) images. Real-time reverse-transcription PCR (RT-PCR) analysis of nasopharyngeal swabs confirmed COVID-19 infection in these patients [5].

#### Encephalopathy

A retrospective study from China reviewed 274 cases of COVID-19, of which 24 (8.8%) developed hypoxic encephalopathy which progressed to death in 23 (95.8%) and recovery in 1 (4.2%) [18]. A separate study from China reported encephalopathy in 8 (2.6%) out of 304 cases, out of which one was obtunded, one was delirious, and six were comatose [40]. Among the deceased cases in both studies, there were high rates of acute respiratory distress syndrome (ARDS), respiratory failure, heart failure, and sepsis.

Three other case reports of COVID-19 encephalopathy have been documented, one each from the USA [15], Iran [17], and Italy [29], with an age group spanning 30–75. All three cases presented with symptoms of fever and cough for a duration of approximately 5 days before the onset of confusion and asthenia. CT and magnetic resonance imaging (MRI) of the brain were unremarkable. Electroencephalogram (EEG) was employed in the USA and Italy case which revealed diffuse slowing consistent with encephalopathy [15, 29]. Chloroquine, lopinavir, and ritonavir were administered in the first two cases. At the time of the report, the first patient was critically ill and the second had complete resolution of her symptoms [15, 17]. The third patient showed dramatic response to high-dose steroids and with slightly more than a week of therapy, he was back at baseline mentation with a normal neurological examination [29].

#### Ischemic stroke

A retrospective observational study from Wuhan, China, reported that six (2.8%) patients, out of the 214 reviewed COVID-19 cases, developed ischemic stroke. Of these six patients, two arrived at the emergency department owing to sudden onset of hemiplegia without any fever or upper respiratory tract symptoms. Five of the six patients were severe cases of COVID-19. D-dimer levels were high in patients with severe disease, compared to non-severe, as well as in those with CNS manifestations, compared to those without [5].

A retrospective observational study from a different center in Wuhan, China, found eleven (5.0%) patients, out of 221 reviewed COVID-19 cases, developed acute ischemic stroke. Those who had COVID-19 infection with new onset of ischemic stroke were more likely to have a severe SARS-CoV-2 presentation, an advanced age (71.6 ± 15.7 years versus 52.1 ± 15.3 years), and pre-existing cardiovascular risk factors including hypertension, diabetes, and previous cerebrovascular disease. An increased inflammatory response and hypercoagulable state, typified by raised CRP and D-dimer levels, was also more common among these patients [7].

Three other cases of ischemic stroke were reported in China among COVID-19 patients aged 40–80. Each of them initially presented with a dry cough. At least 1 week later, they developed limb weakness, dysarthria, and tongue deviation. A CT scan of the brain revealed basal ganglia infarction in one case; normal findings were reported in the other two cases. Laboratory studies showed elevated D-dimer, decreased fibrinogen level, prolonged prothrombin time (PT), and activated partial thromboplastin time (APTT). Additionally, serum cytokine levels including IL-6, IL-8, and IL-10, were markedly elevated. All three patients recovered with good functional outcomes after stroke treatment and supportive therapy [13, 26].

Oxley et al. reported five cases of large-vessel stroke in patients younger than 50 years of age from the USA. Only three cases were symptomatic for COVID-19. The mean NIHSS score was 17 at admission. CT brain, CT angiography, and/or MRI revealed severe large-vessel stroke in all patients. All but one underwent mechanical thrombectomy. Among these patients, D-dimer, ferritin, PT, and APTT were raised while the fibrinogen level was reduced. One was discharged home, two were transferred to a rehabilitation facility, and two were in the ICU/stroke unit at the time of the report [39].

#### Intracerebral hemorrhage

A retrospective observational study from Wuhan, China, reported one (0.45%) patient, out of 221 reviewed COVID-19 cases, who developed intracerebral hemorrhage. He was a 62-year-old male cigarette smoker who presented to the hospital with a severe case of COVID-19. About a week later, he developed an intracranial hemorrhage and died. Of note, his blood pressure averaged 150/80 mmHg during treatment [7].

There are two case reports from Iran, one of a 79-year-old male with no medical history of hypertension or anticoagulation therapy [14] and another of a 54-year-old lady with a history of hypertension [38]. Both presented with an acute loss of consciousness preceded by fever and dry cough. Initial Glasgow Coma Scale (GCS) for them was seven and ten, respectively. Coagulation studies were normal for both. The CT brain revealed massive intracerebral hemorrhage in the right hemisphere accompanied by intraventricular and subarachnoid hemorrhage for the former, and bilateral sub-acute basal ganglia hemorrhage in the latter, and a lung CT scan showed bilateral ground-glass opacities in both cases. The respective authors suspected the intracranial hemorrhage was secondary to infection with SARS-CoV-2 in both cases.

#### Encephalitis and encephalomyelitis

Six cases of encephalitis have been described to date; two from China [12, 27], two from Switzerland [31], one from Japan [24], and one from America [11]. Their ages range from early 20s to late 60s. All of them had preceding symptoms of fever and cough followed by a rapidly deteriorating level of consciousness. Meningeal irritability in the form of nuchal rigidity, Kernig’s, and Brudzinski’s was reported in two out of the six cases [24, 27].

Where lumbar puncture (LP) was performed, it showed lymphocytic pleocytosis typical of a viral meningo-encephalitis [12, 24, 31]. SARS-CoV-2 was detected in the cerebrospinal fluid (CSF) in only one Chinese patient [12] and Japanese patient [24]. For one of the Switzerland patients who presented with psychosis and focal status epilepticus, an EEG was done which showed abundant bursts of anterior low-medium voltage irregular spike and waves superimposed on an irregularly slowed theta background. This was managed with intravenous (IV) clobazam and valproate, and she had a marked improvement 96 h after admission with the resolution of her symptoms [31].

CT brain was normal in all but the case of the American whose scan demonstrated symmetric hypoattenuation within the bilateral medial thalami. She had a follow-up MRI brain which revealed hemorrhagic, rim-enhancing lesions within the bilateral thalami, medial temporal lobes, and subinsular regions. In her case, a presumptive diagnosis of COVID-19-associated acute necrotizing hemorrhagic encephalopathy was made, and she was thereafter started on intravenous immunoglobulin (IVIG) [11]. The MRI of the patient from Japan revealed right lateral ventriculitis and encephalitis mainly involving the mesial temporal lobe and hippocampus [24]. MRI brain performed for the Switzerland patients were normal [31]. At the time of writing, the Chinese and Switzerland patients recovered completely with supportive therapy [12, 24, 27], and the outcome of the patients from America and Japan is not known.

One case of acute disseminated encephalomyelitis (ADEM) has been reported in New Jersey, America [30]. The case was a female in her early 40s who presented with a 2-day history of dysphagia, dysarthria, and encephalopathy preceded by headache and myalgia. COVID-19 nasal swab RT-PCR was positive. A lumbar puncture (LP) showed a CSF with normal cell counts, protein, and glucose. CT brain showed multifocal patchy areas of white matter hypoattenuation and MRI brain showed extensive patchy areas involving bilateral frontoparietal white matter, anterior temporal lobes, basal ganglia, external capsules, and thalami. She was treated with hydroxychloroquine, ceftriaxone, and a 5-day course of IVIG. Improvement was noted at the 5-day mark of therapy.

#### Acute myelitis

One COVID-19 case from Wuhan, China, came with the presentation of acute myelitis. A 66-year-old male, with a 5-day history of fever, was admitted to the hospital after outpatient oral moxifloxacin hydrochloride and oseltamivir failed to improve his symptoms. CT scan of the chest found patchy changes in both lungs, and nasopharyngeal swab tested positive for SARS-CoV-2. After a night of high fever, he developed bilateral weakness of his lower limbs, with urinary and bowel incontinence, rapidly progressing to flaccid lower extremity paralysis and paresthesia and numbness below T10. Planters were down going bilaterally. A clinical diagnosis of post-infectious acute myelitis was made. He received treatment with ganciclovir, lopinavir/ritonavir, moxifloxacin, dexamethasone, IVIG, and mecobalamin. His bilateral lower extremity paralysis ultimately improved, and he was discharged to a rehabilitation facility [16].

### Peripheral nervous system manifestations

#### Loss of smell/taste sensation

Five studies assessed the prevalence of smell disorder. An overall average of 59.45% of patients experienced olfactory disturbance (Fig. 2). Additionally, four studies assessed the prevalence of taste disorder. An overall average of 56.48% of patients experienced taste disorder (Fig. 2).

A multicenter prospective study by Lechien et al., reported time course of the aforementioned disorders. The olfactory dysfunction appeared before, in unison, and after the appearance of general symptoms in 11.8, 22.8, and 65.4% of the cases, respectively. This olfactory abnormality persisted after the resolution of other symptoms in 63.0% of cases [36]. In an Iranian study by Moein et al., majority of patients with olfactory dysfunction reported that the onset of the olfactory dysfunction occurred at the same time or immediately after the onset of their other COVID-19 symptoms [37].

#### Bell’s palsy

In China, a 65-year-old woman was admitted to the hospital for a left facial droop preceded by a 2-day history of pain in the mastoid region. There was no preceding fever, cough, or respiratory symptoms. Physical examination showed a left lower motor neuron facial nerve paralysis. MRI brain showed no abnormality. However, a throat swab RT-PCR turned positive for SARS-CoV-2 virus and the CT chest revealed ground-glass shadows in the right lower lung. The left facial paralysis was relieved after antiviral treatment with umifenovir and ribavirin [28].

#### Guillain-Barré syndrome and its variants

Toscano et al. reported 5 cases of Guillain-Barré syndrome (GBS) in COVID-19 patients. The interval between the onset of viral illness and the first symptoms of GBS was between 5 and 10 days. The first symptoms of GBS were lower limb weakness and paresthesia in four patients and facial diplegia followed by ataxia and paresthesia in one patient. Generalized, flaccid quadriparesis or tetraplegia evolved over a period of 36 h to 4 days in four patients; three received mechanical ventilation. CSF analysis showed raised protein levels in two patients and raised lymphocytes in all five patients. CSF RT-PCR for COVID-19 was negative in all patients. Neurological examination and nerve conduction studies were consistent with a demyelinating neuropathy in two patients, while the others were diagnosed as axonal variants of GBS. All five patients were treated with IVIG; two received a second course of IVIG and one required plasma exchange. Four weeks after treatment, two patients remained in the ICU and were receiving mechanical ventilation, two were undergoing physical therapy, and one was discharged without any residual symptoms [8].

Four other case reports of GBS, one in America [34], Iran [33], Italy [32], and China [9], respectively, were reported. Age ranged from mid-50s to 70s. All but the Chinese patient presented with flu-like symptoms preceding weakness and/or numbness of the lower limbs with a progressive ascending paralysis; the Chinese patient presented with neurological symptoms first and respiratory symptoms only on day 8 of admission [9]. In all cases, neurological examination and nerve conduction studies were consistent with a demyelinating neuropathy [9, 32–34]. Where LP was done, an albuminocytologic dissociation was revealed [9, 32]. IVIG was administered in the above 4 case reports with variable response.

Two GBS variants have been described in a case series from Spain. Specifically, one case of Miller Fisher syndrome and one case of polyneuritis cranialis associated with COVID-19 was described. The former case was a 50-year-old man who presented with anosmia, ageusia, right internuclear ophthalmoparesis, right fascicular oculomotor palsy, ataxia, areflexia, albuminocytologic dissociation, and positive testing for GD1b-IgG antibodies. Five days prior, he had developed a cough, malaise, headache, low back pain, and a fever. The latter case was a 39-year-old man who presented with ageusia, bilateral abducens palsy, areflexia, and albuminocytologic dissociation. Three days prior, he had developed diarrhea, a low-grade fever, and a poor general condition. The oropharyngeal swab for COVID-19 by RT-PCR was positive in both patients and negative in the cerebrospinal fluid. The first patient was treated with IVIG and the second with acetaminophen. Two weeks later, both patients made a complete neurological recovery, except for residual anosmia and ageusia in the first case [10].

### Skeletal muscle manifestations

#### Myalgia and myositis

An overall average of 25.1% of patients reported experiencing myalgia in seven retrospective studies (Fig. 2). The character and course of myalgia was not detailed in any of those studies.

Mao et al. reported that out of 214 COVID-19 patients, skeletal muscle injury occurred in 23 (10.7%). Compared with the patients without muscle injury, patients with muscle injury had significantly higher levels of creatine kinase (median 400 U/L [range 203.0 to 12,216.0] versus median 58.5 U/L [range 8.8–212]; *P* < 0.001). In addition, patients with muscle injury had higher C-reactive protein (CRP) levels and D-dimer levels—manifestations of increased inflammatory response and associated coagulopathy. Furthermore, patients with muscle injury showed more signs of multi-organ damage, including more serious liver and kidney abnormalities, than patients without muscle injury [5].

#### Rhabdomyolysis

A case report by Jin et al. described a 60-year-old man in Wuhan who presented with a 6-day history of fever and cough and a CT chest which showed bilateral ground-glass opacities. RT-PCR analysis of the patient’s throat swab specimen indicated SARS-CoV-2 infection. He was treated supportively and with antivirals and antibiotics. On day 9 of admission, the patient complained of pain and weakness in his lower limbs, and tenderness was noted on examination. Urgent laboratory reports indicated raised myoglobin (> 12,000.0 μg/L), creatine kinase (11,842 U/L), lactate dehydrogenase (2347 U/L), alanine aminotransferase (111 U/L), and aspartate aminotransferase (213 U/L). The patient’s kidney function and electrolytes were normal. Urinalysis revealed a light-yellow color of the urine, with positive occult blood and positive urine protein. These results indicated the onset of rhabdomyolysis. In addition to ongoing supportive therapy, the patient was treated with hydration, alkalization, plasma transfusion, and gamma globulin. The patient improved subsequently [25].

## Discussion

Coronavirus contains four structural proteins, including the spike protein (S), envelope protein (E), membrane protein (M), and the nucleocapsid protein (N). Among them, the S protein plays the most important role in virus attachment, fusion, and entry. SARS-CoV-2, like SARS-CoV, recognizes the angiotensin-converting enzyme 2 (ACE2) as a host cell entry receptor [41]. High expression of the ACE2 receptor is seen in type II alveolar cells of the lung, intestine, esophagus, cardiomyocytes, proximal tubular cells, and urothelial cells [42]. Glial cells and neurons have been reported to express ACE2 receptors, making the brain a potential target of COVID-19 infection [43].

How the virus enters the central nervous system is still a subject of debate. One plausible route of entry is through the olfactory nerve. Retrograde transfer into the axon, whether through synapses, endocytosis, or exocytosis, could explain viral migration into the brain [44, 45]. Experimental studies using transgenic mice suggest that SARS-CoV and MERS-COV may enter the central nervous system intranasally through the olfactory nerve. Once in the brain, they have the potential to spread to the thalamus and brain stem, two regions highly involved in coronaviridae infections [46–48]. A study evaluating specific receptors in the nasal mucosa, however, suggest SARS-CoV-2 may reach the brain through mechanisms independent of axonal transport via olfactory sensory nerves [49].

Another theory suggests if SARS-CoV-2 gained access to the general circulation, it could potentially invade the cerebral circulation and continue viral spread [43]. Slow movement in the cerebral microvasculature may promote interaction of the SARS-CoV-2 S protein and capillary endothelium ACE2 receptor. Once bound, the virus would have the potential to infect, damage, and bud from the capillary endothelium, thereby facilitating viral entry into the cerebrum [43, 50].

SARS-CoV-2 could also cause damage to the central nervous system indirectly. Viruses do not have to enter the brain to cause damage; they can activate an immune response that triggers subsequent damage within neuronal tissue. SARS-CoV-2 has been reported to cause a massive release of cytokines, a syndrome known as “cytokine storm”—downstream effects of this immune response include endothelial damage, disseminated intravascular coagulation, and disrupted cerebral auto-regulation [51, 52].

Common symptoms elucidated from our systematic review include myalgia, headache, altered sensorium, hyposmia, and hypogeusia. Acute viral respiratory tract infections have already been shown to cause the aforementioned neurological symptoms [53]. Peri- and post-infectious hyposmia and hypogeusia is hypothesized to be secondary to olfactory nerve and/or apparatus damage from direct insult of viral infection [54]. According to the multicenter prospective European study by Lechien et al., it appears that a large number of COVID-19 patients experience olfactory and gustatory symptoms. In their study, olfactory and gustatory dysfunction occurred in 85.6 and 88.8% of patients, respectively, with a majority of patients (65.4%) experiencing abnormalities in olfaction after the appearance of their general symptoms [36].

We found that in COVID-19 patients, altered sensorium and encephalopathy were not uncommon. The basic pathological change seen in this disease is cerebral edema, with key clinical features being headache, confusion, delirium, loss of consciousness, seizure, and coma. COVID-19 patients with encephalopathy were, by large, older male patients with cardiovascular comorbidities and severe infection with systematic inflammation and multi-organ dysfunction. Early identification of COVID-19 patients with altered sensorium is critical, as underlying potential reversible causes, including impending respiratory failure, require timely intervention [55]. The pathophysiology behind the cerebral dysfunction is hypothesized to be in part inflammatory-mediated [56]. This is supported by the fact that the encephalopathic Italian patient had a dramatic response to high-dose steroids [29].

Ischemic stroke is another clinical entity which can present in patients with COVID-19 infection. This presentation may arise secondary to a cytokine storm syndrome [51], which can cause endothelial damage, disseminated intravascular coagulation, and disrupted cerebral auto-regulation [52]. Through the ACE2 receptor of the vascular endothelium, the virus's extensive invasion of the vascular endothelium obviously may cause extensive endotheliitis, increasing the risk of thrombosis leading to ischemic stroke. Critically ill patients with severe SARS-CoV-2 infection often show elevated levels of D-dimer, a fibrin-degraded product which serves as a marker of dysfunctional activation of the coagulation system, such as in acute ischemic stroke [1, 5, 57]. However, it is observed that the stroke prevalence in COVID-19 patients and non-COVID-19 patients are similar. Two retrospective studies in Wuhan, China, reported prevalence rates of between 2.8–5.0% of acute ischemic stroke [5, 7] which is consistent with the stroke prevalence in Northeast China prior to the outbreak [58, 59]. Although the biochemistry suggests an association of COVID-19 with disorders of the coagulation system, further studies are warranted to establish a relationship.

For the three cases of COVID-19-associated intracerebral hemorrhage, a few potential explanations exist. It is known that the expression and the ability of ACE2 receptor to lower blood pressure is reduced in patients with hypertension. In SARS-CoV-2 infection, the presence of S protein could further reduce the expression and function of ACE2 proteins. This could potentially lead to uncontrolled hypertension, arterial wall rupture, and cerebral hemorrhage in infected patients [56]. Moreover, if the virus disseminates within the cerebral microvasculature, subsequent damage of capillary endothelial cells could result in a tear of the vasculature sufficient enough to cause parenchymal hemorrhage [43]. Additionally, COVID-19 patients have been reported to have thrombocytopenia and coagulopathy, both of which are contributory factors for secondary brain parenchymal hemorrhage [5, 60].

It is still unknown how COVID-19 causes encephalo-myelitis. It is thought that once viral particles gain entry into the milieu of the neuronal tissue, their interaction with the ACE2 receptors in neurons could initiate a cycle of viral budding accompanied by neuronal damage. This can occur without substantial inflammation [43]. In other cases, it may cause an acute inflammatory demyelination resulting in ADEM, which was described in one COVID-19 case [30], and previously in MERS-CoV [61]. COVID-19 is also thought to cause acute hemorrhagic encephalitis through the mechanism of a cytokine storm [11]. Acute hemorrhagic leukoencephalitis is a rare demyelinating disorder that is usually fatal; ICU care, use of high-dose corticosteroid therapy, immunoglobulins, plasma exchange, and dehydrating agents have led to survival in only some patients [62]. This has been seen in one COVID-19 case thus far [11].

Three neuro-immunological entities related to COVID-19 infection surfaced in our systematic review: acute transverse myelitis, GBS and its variants, and Bell’s palsy [9, 16, 28]. The time course of the disease is alike what is known for other viruses known to cause the above in that the symptoms of respiratory or gastrointestinal infection usually precede that of the neuro-immunological phenomena [63]. This was seen in all cases in this report with the only exception being the GBS case report from China [9]. This suggests that the underlying mechanism of such neuro-immunological phenomena in COVID-19 patients is likely to be grounded by the hypothesis of molecular mimicry, where mimicry between microbial and nerve antigens is thought to be a major driving force behind the development of the disorder [63].

There are a number of limitations in our study. Most of the studies included in this systematic review are case reports/series and retrospective observational studies. The larger retrospective studies included were limited to the documentation of common neurological symptoms, such as headache, myalgia, and a loss in sense of taste and/or smell. Furthermore, the timeliness of our systematic review presents potential for premature analysis of data trends in the literature. Given the current pandemic, the authors of this study felt that the benefits of systematic data retrieval and review outweighed current risks of inaccurate predications. Our aim was to synthesize data to aid physicians currently treating the novel COVID-19, knowing time constraints inhibited their own analysis of the evolving literature on neurological manifestations.

## Conclusion

Our systematic review comprehensively detailed the neurological manifestations of COVID-19 known to date. Respiratory symptoms remain the hallmark of early identification, cohorting, and treatment of COVID-19, bearing in mind that in a number of cases it has been observed that neurological manifestations may precede it in the course of the disease.

## Data Availability

Not applicable.

## References

[1] Guan WJ, Ni ZY, Hu Y, Liang WH, Ou CQ, He JX, Liu L, Shan H, Lei CL, Hui DS, Du B. Clinical characteristics of coronavirus disease 2019 in China. N Engl J Med. 2020;382(18):1708–20. 10.1056/NEJMoa2002032 PMC7092819 32109013

[2] Huang C, Wang Y, Li X, Ren L, Zhao J, Hu Y (2020). Clinical features of patients infected with 2019 novel coronavirus in Wuhan, China. Lancet.

[3] Bhatraju PK, Ghassemieh BJ, Nichols M, Kim R, Jerome KR, Nalla AK, Greninger AL, Pipavath S, Wurfel MM, Evans L, Kritek PA. Covid-19 in critically ill patients in the Seattle region—case series. N Engl J Med. 2020;382(21):2012–22. 10.1056/NEJMoa2004500 PMC7143164 32227758

[4] Nath A. Neurologic complications of coronavirus infections. Neurology. 2020;94(19):809–10. 10.1212/WNL.0000000000009455 32229625

[5] Mao L, Jin H, Wang M, Hu Y, Chen S, He Q, Chang J, Hong C, Zhou Y, Wang D, Miao X. Neurologic manifestations of hospitalized patients with coronavirus disease 2019 in Wuhan, China. JAMA Neurol. 2020;77(6):683–90. 10.1001/jamaneurol.2020.1127 PMC7149362 32275288

[6] Liberati A, Altman DG, Tetzlaff J, Mulrow C, Gøtzsche PC, Ioannidis JPA (2009). The PRISMA statement for reporting systematic reviews and meta-analyses of studies that evaluate health care interventions: explanation and elaboration. J Clin Epidemiol.

[7] Li Y, Wang M, Zhou Y, Chang J. Acute cerebrovascular disease following COVID-19: a single center, retrospective, observational study. SSRN Electron J. 2020. Available at https://ssrn.com/abstract=3550025. Accessed 3 Mar 2020. 10.1136/svn-2020-000431 PMC7371480 32616524

[8] Toscano G, Palmerini F, Ravaglia S, et al. Guillain-Barré Syndrome Associated with SARS-CoV-2. N Engl J Med. 2020;382(26):2574–76. 10.1056/nejmc2009191. 10.1056/NEJMc2009191 PMC7182017 32302082

[9] Zhao H, Shen D, Zhou H, Liu J, Chen S (2020). Guillain-Barré syndrome associated with SARS-CoV-2 infection: causality or coincidence?. Lancet Neurol.

[10] Gutiérrez-Ortiz C, Méndez A, Rodrigo-Rey S, et al. Miller Fisher Syndrome and polyneuritis cranialis in COVID-19. Neurology. 2020. 10.1212/wnl.0000000000009619. . 10.1212/WNL.0000000000009619 32303650

[11] Poyiadji N, Shahin G, Noujaim D, Stone M, Patel S, Griffith B. COVID-19–associated acute hemorrhagic necrotizing encephalopathy: CT and MRI features. Radiology. 2020;1:201187. 10.1148/radiol.2020201187 PMC7233386 32228363

[12] Huaxia. Beijing hospital confirms nervous system infections by novel coronavirus. Xinhua News. 2020; Available from: http://www.xinhuanet.com/english/2020-03/05/c_138846529.htm. Accessed 18 Apr .

[13] Pan Zhai, Yanbing Ding, Yiming Li et al. The impact of COVID-19 on ischemic stroke: A case report, 31 March 2020, PREPRINT (Version 1) available at Research Square [10.21203/rs.3.rs-20393/v1]. 10.1186/s13000-020-00994-0 PMC7323364 32600350

[14] Karimi N, Rouhani N. COVID 19 and intra cerebral hemorrhage: causative or coincidental. New Microbes New Infect. 2020:100669. 10.1016/j.nmni.2020.100669. 10.1016/j.nmni.2020.100669 PMC7163302 32322398

[15] Filatov A, Sharma P, Hindi F, Espinosa PS. Neurological Complications of Coronavirus Disease (COVID-19): Encephalopathy. Cureus. 2020;12(3):e7352. 10.7759/cureus.7352. 10.7759/cureus.7352 PMC7170017 32328364

[16] Zhao K, Huang J, Dai D, et al. Acute myelitis after SARS-CoV-2 infection: a case report. medRxiv; 2020. 10.1101/2020.03.16.20035105.

[17] Karimi N, Razavi AS, Rouhani N (2020). Frequent convulsive seizures in an adult patient with COVID-19 : a case report. Iran Red Crescent Med J.

[18] Chen T, Di Wu HC, Yan W, Yang D, Chen G, Ma K, Xu D, Yu H, Wang H, Wang T, Guo W. Clinical characteristics of 113 deceased patients with coronavirus disease 2019: retrospective study. BMJ. 2020;368. 10.1136/bmj.m1091 PMC7190011 32217556

[19] Qiu H, Wu J, Hong L, Luo Y, Song Q, Chen D (2019). Clinical and epidemiological features of 36 children with coronavirus disease 2019 (COVID-19) in Zhejiang, China: an observational cohort study. Lancet Infect Dis.

[20] Zhou F, Yu T, Du R, Fan G, Liu Y, Liu Z (2020). Clinical course and risk factors for mortality of adult inpatients with COVID-19 in Wuhan, China: a retrospective cohort study. Lancet.

[21] Chen N, Zhou M, Dong X, Qu J, Gong F, Han Y (2020). Epidemiological and clinical characteristics of 99 cases of 2019 novel coronavirus pneumonia in Wuhan, China: a descriptive study. Lancet.

[22] Wang D, Hu B, Hu C, Zhu F, Liu X, Zhang J, Wang B, Xiang H, Cheng Z, Xiong Y, Zhao Y. Clinical characteristics of 138 hospitalized patients with 2019 novel coronavirus–infected pneumonia in Wuhan, China. Jama. 2020;323(11):1061–9. 10.1001/jama.2020.1585 PMC7042881 32031570

[23] Giacomelli A, Pezzati L, Conti F, et al. Self-reported olfactory and taste disorders in SARS-CoV-2 patients: a cross-sectional study. Clinical Infectious Diseases: an Official Publication of the Infectious Diseases Society of America. 2020. 10.1093/cid/ciaa330. 10.1093/cid/ciaa330 PMC7184514 32215618

[24] Moriguchi T, Harii N, Goto J, et al. A first case of meningitis/encephalitis associated with SARS-Coronavirus-2. International Journal of Infectious Diseases: IJID : Official Publication of the International Society for Infectious Diseases. 2020;94:55–58. 10.1016/j.ijid.2020.03.062. 10.1016/j.ijid.2020.03.062 PMC7195378 32251791

[25] Jin M, Tong Q. Rhabdomyolysis as Potential Late Complication Associated with COVID-19. Emerg Infect Dis. 2020;26(7):1618–20. 10.3201/eid2607.200445. 10.3201/eid2607.200445 PMC7323559 32197060

[26] Bin Fu, Yun Chen, Ping Li et al. The 2019 novel coronavirus disease with secondary ischemic stroke: two cases report, 05 April 2020, PREPRINT (Version 1) available at Research Square [10.21203/rs.3.rs-20943/v1]. 10.1186/s12883-020-02033-3 PMC7781816 33397310

[27] Ye M, Ren Y, Lv T. Encephalitis as a clinical manifestation of COVID-19. Brain, Behavior, and Immunity. 2020. 10.1016/j.bbi.2020.04.017. 10.1016/j.bbi.2020.04.017 PMC7146652 32283294

[28] Yue Wan, Shugang Cao, Qi Fang et al. Coronavirus disease 2019 complicated with Bell’s palsy: a case report, 16 April 2020, PREPRINT (Version 1) available at Research Square [10.21203/rs.3.rs-23216/v1].

[29] Pilotto A, Odolini S, S SM, Comelli A, Gazzina S, Psy SN, et al. Steroid-responsive severe encephalopathy in SARS-CoV-2 infection. medRxiv Preprint. 2020. Available at 10.1101/2020.04.12.20062646.

[30] Zhang T, Rodricks MB, Hirsh E, Wood R, Somerset H, Ave R. COVID-19-associated acute disseminated encephalomyelitis – a case report. medRxiv Preprint. 2020. Available at 10.1101/2020.04.16.20068148.

[31] Bernard-valnet R, Pizzarotti B, Anichini A. Two patients with acute meningo-encephalitis concomitant to SARS-CoV-2 infection. medRxiv Preprint. 2020. Available at 10.1101/2020.04.17.20060251.

[32] Padroni M, Mastrangelo V, Asioli GM, et al. Guillain-Barré syndrome following COVID-19: new infection, old complication? J Neurol. 2020;267(7):1877–79. 10.1007/s00415-020-09849-6. 10.1007/s00415-020-09849-6 PMC7180646 32333166

[33] Sedaghat Z, Karimi N. Guillain Barre syndrome associated with COVID-19 infection: A case report. J Clin Neurosci. 2020;76:233–35. 10.1016/j.jocn.2020.04.062. 10.1016/j.jocn.2020.04.062 PMC7158817 32312628

[34] Virani A, Rabold E, Hanson T, Haag A, Elrufay R, Cheema T, Balaan M, Bhanot N. Guillain-Barré syndrome associated with SARS-CoV-2 infection. IDCases. 2020;18:e00771. 10.1016/j.idcr.2020.e00771 PMC7165113 32313807

[35] Yan CH, Faraji F, Prajapati DP, Ostrander BT, DeConde AS. Self-reported olfactory loss associates with outpatient clinical course in COVID-19. Int Forum Allergy Rhinol. 2020;10(7):821–31. 10.1002/alr.22592. 10.1002/alr.22592 PMC7264572 32329222

[36] Lechien JR, Chiesa-Estomba CM, De Siati DR, Horoi M, Le Bon SD, Rodriguez A, Dequanter D, et al. Olfactory and gustatory dysfunctions as a clinical presentation of mild-to-moderate forms of the coronavirus disease (COVID-19): a multicenter European study. Eur Arch Otorhinolaryngol. 2020;6:1–11. 10.1007/s00405-020-05965-1. Epub ahead of print. 10.1007/s00405-020-05965-1 PMC7134551 32253535

[37] Moein ST, Hashemian SM, Mansourafshar B, Khorram-Tousi A, Tabarsi P, Doty RL. Smell dysfunction: a biomarker for COVID-19. Int Forum Allergy Rhinol. 2020;17: 10.1002/alr.22587. Epub ahead of print. . 10.1002/alr.22587 PMC7262123 32301284

[38] Haddadi K, Ghasemian R, Shafizad M. Basal Ganglia Involvement and Altered Mental Status: A Unique Neurological Manifestation of Coronavirus Disease 2019. Cureus. 2020;12(4):e7869. 10.7759/cureus.7869. 10.7759/cureus.7869 PMC7255547 32489724

[39] Oxley TJ, Mocco J, Majidi S, Kellner CP, Shoirah H, Singh IP, De Leacy RA, Shigematsu T, Ladner TR, Yaeger KA, Skliut M. Large-vessel stroke as a presenting feature of Covid-19 in the young. N Eng J Med. 2020;382(20):e60. 10.1056/NEJMc2009787 PMC7207073 32343504

[40] Lu L, Xiong W, Liu D, Liu J, Yang D, Li N, Mu J, Guo J, Li W, Wang G, Gao H, Zhang Y, Lin M, Chen L, Shen S, Zhang H, Sander JW, Luo J, Chen S, Zhou D. New onset acute symptomatic seizure and risk factors in coronavirus disease 2019: A retrospective multicenter study. Epilepsia. 2020;61(6):e49–e53. 10.1111/epi.16524. 10.1111/epi.16524 PMC7264627 32304092

[41] Tai W, He L, Zhang X, Pu J, Voronin D, Jiang S, Zhou Y, Du L. Characterization of the receptor-binding domain (RBD) of 2019 novel coronavirus: implication for development of RBD protein as a viral attachment inhibitor and vaccine. Cell Mol Immunol. 2020;17(6):613–20. 10.1038/s41423-020-0400-4 PMC7091888 32203189

[42] Zhang H, Penninger JM, Li Y, Zhong N, Slutsky AS (2020). Angiotensin-converting enzyme 2 (ACE2) as a SARS-CoV-2 receptor: molecular mechanisms and potential therapeutic target. Intensive Care Med.

[43] Baig AM, Khaleeq A, Ali U, Syeda H. Evidence of the COVID-19 virus targeting the CNS: tissue distribution, host–virus interaction, and proposed neurotropic mechanisms. ACS Chem Neurosci. 2020;11(7):995–8. 10.1021/acschemneuro.0c00122 32167747

[44] Dubé M, Le Coupanec A, Wong AHM, Rini JM, Desforges M, Talbot PJ (2018). Axonal transport enables neuron-to-neuron propagation of human coronavirus OC43. J Virol.

[45] Munster VJ, Prescott JB, Bushmaker T, Long D, Rosenke R, Thomas T (2012). Rapid Nipah virus entry into the central nervous system of hamsters via the olfactory route. Sci Rep.

[46] McCray PB, Pewe L, Wohlford-Lenane C, Hickey M, Manzel L, Shi L (2007). Lethal infection of K18-hACE2 mice infected with severe acute respiratory syndrome coronavirus. J Virol.

[47] Li K, Wohlford-Lenane C, Perlman S, Zhao J, Jewell AK, Reznikov LR (2015). Middle east respiratory syndrome coronavirus causes multiple organ damage and lethal disease in mice transgenic for human dipeptidyl peptidase 4. J Infect Dis.

[48] Netland J, Meyerholz DK, Moore S, Cassell M, Perlman S (2008). Severe acute respiratory syndrome coronavirus infection causes neuronal death in the absence of encephalitis in mice transgenic for human ACE2. J Virol.

[49] Brann DH, Tsukahara T, Weinreb C, Logan DW, Datta SR. Non-neural expression of SARS-CoV-2 entry genes in the olfactory epithelium suggests mechanisms underlying anosmia in COVID-19 patients. bioRxiv Preprint. 2020. Available at 10.1101/2020.03.25.009084.

[50] Conde Cardona G, Quintana Pájaro LD, Quintero Marzola ID, Ramos Villegas Y, Moscote Salazar LR. Neurotropism of SARS-CoV 2: Mechanisms and manifestations. J Neurol Sci. 2020;412:116824. 10.1016/j.jns.2020.116824. 10.1016/j.jns.2020.116824 PMC7141641 32299010

[51] Monteleone G, Sarzi-Puttini PC, Ardizzone S. Preventing COVID-19-induced pneumonia with anticytokine therapy. Lancet Rheumatol. 2020;2(5):e255–6. 10.1016/S2665-9913(20)30092-8 PMC7193140 32368737

[52] Jenny NS, Callas PW, Judd SE, McClure LA, Kissela B, Zakai NA (2019). Inflammatory cytokines and ischemic stroke risk: the REGARDS cohort. Neurology..

[53] E. Kuchar, K. Mis’kiewicz, Aneta Nitsch-Osuch and LS. Pathophysiology of clinical symptoms in acute viral respiratory tract infections. Advs Exp Med Biol Respir. 2015;12:25–38. 10.1007/5584_2015_110 PMC7121097 25786400

[54] Henkin RI, Larson AL, Powell RD (2015). Hypogeusia, dysgeusia, hyposmia, and dysosmia following influenza-like infection. Ann Otol.

[55] Kotfis K, Williams Roberson S, Wilson JE, et al. COVID-19: ICU delirium management during SARS-CoV-2 pandemic. Crit Care. 2020;24(1):176. 10.1186/s13054-020-02882-x. 10.1186/s13054-020-02882-x PMC7186945 32345343

[56] Wu Y, Xu X, Chen Z, et al. Nervous system involvement after infection with COVID-19 and other coronaviruses. Brain, Behavior, and Immunity. 2020;87:18–22. 10.1016/j.bbi.2020.03.031. 10.1016/j.bbi.2020.03.031 PMC7146689 32240762

[57] Alhazzani W, Møller MH, Arabi YM, et al. Surviving Sepsis Campaign: guidelines on the management of critically ill adults with Coronavirus Disease 2019 (COVID-19). Intensive Care Med. 2020;46(5):854–87. 10.1007/s00134-020-06022-5. 10.1007/s00134-020-06022-5 PMC7101866 32222812

[58] Zhang FL, Guo ZN, Wu YH, Liu HY, Luo Y, Sun MS (2017). Prevalence of stroke and associated risk factors: a population based cross sectional study from Northeast China. BMJ Open.

[59] Wang W, Jiang B, Sun H, Ru X, Sun D, Wang L (2017). Prevalence, incidence, and mortality of stroke in China: results from a nationwide population-based survey of 480 687 adults. Circulation..

[60] Zhang Y, Xiao M, Zhang S, Xia P, Cao W, Jiang W, Chen H, Ding X, Zhao H, Zhang H, Wang C. Coagulopathy and antiphospholipid antibodies in patients with Covid-19. New England J Med. 2020;382(17):e38. 10.1056/NEJMc2007575 PMC7161262 32268022

[61] Arabi YM, Harthi A, Hussein J, Bouchama A, Johani S, Hajeer AH (2015). Severe neurologic syndrome associated with Middle East respiratory syndrome corona virus (MERS-CoV). Infection.

[62] Chellathurai A, Ponnusamy S, Periakaruppan A, Gopinathan K, Philson J (2016). Acute hemorrhagic leucoencephalitis. Indian J Pediatr.

[63] Willison HJ, Jacobs BC, van Doorn PA (2016). Guillain-Barré syndrome. Lancet..

